# Comparative genomic sequence analysis of strawberry and other rosids reveals significant microsynteny

**DOI:** 10.1186/1756-0500-3-168

**Published:** 2010-06-16

**Authors:** Sook Jung, Ilhyung Cho, Bryon Sosinski, Albert Abbott, Dorrie Main

**Affiliations:** 1 Department of Horticulture and Landscape Architecture, Washington State University, Pullman, WA 99164, USA; 2 Computer Science Department, Saginaw Valley State University, University Center, MI 48710, USA; 3 Department of Horticultural Science, North Carolina State University, Raleigh, NC 27695, USA; 4 Department of Genetics and Biochemistry, Clemson University, Clemson, SC 29634, USA

## Abstract

**Background:**

*Fragaria *belongs to the Rosaceae, an economically important family that includes a number of important fruit producing genera such as *Malus *and *Prunus*. Using genomic sequences from 50 *Fragaria *fosmids, we have examined the microsynteny between *Fragaria *and other plant models.

**Results:**

In more than half of the strawberry fosmids, we found syntenic regions that are conserved in *Populus*, *Vitis*, *Medicago *and/or *Arabidopsis *with *Populus *containing the greatest number of syntenic regions with *Fragaria*. The longest syntenic region was between LG VIII of the poplar genome and the strawberry fosmid 72E18, where seven out of twelve predicted genes were collinear. We also observed an unexpectedly high level of conserved synteny between *Fragaria *(rosid I) and *Vitis *(basal rosid). One of the strawberry fosmids, 34E24, contained a cluster of R gene analogs (RGAs) with NBS and LRR domains. We detected clusters of RGAs with high sequence similarity to those in 34E24 in all the genomes compared. In the phylogenetic tree we have generated, all the NBS-LRR genes grouped together with *Arabidopsis *CNL-A type NBS-LRR genes. The *Fragaria *RGA grouped together with those of *Vitis *and *Populus *in the phylogenetic tree.

**Conclusions:**

Our analysis shows considerable microsynteny between *Fragaria *and other plant genomes such as *Populus*, *Medicago*, *Vitis*, and *Arabidopsis *to a lesser degree. We also detected a cluster of NBS-LRR type genes that are conserved in all the genomes compared.

## Background

**Figure 1:**
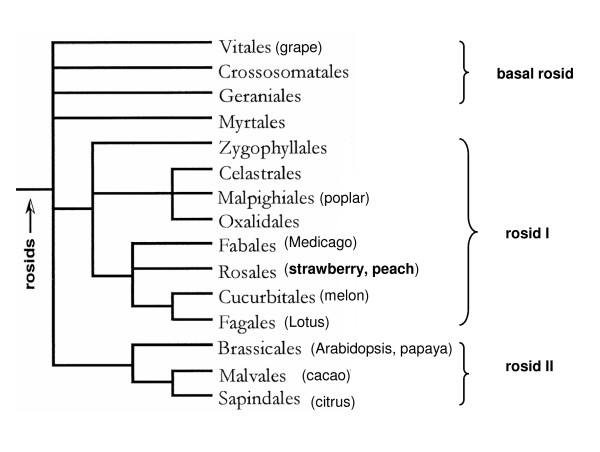
**Phylogenetic relationship of rosids**. The figure is a modified version of Figure 1 in Judd and Olmstead 2004 [30].

Genetic mapping with common markers has indicated significant synteny in many plant families, including Rosaceae [1,2]. These studies have suggested that there is a significant conserved synteny among closely related plant genomes and the degree of synteny decreases with evolutionary distance. Comparative DNA analyses, however, has shown that large segmental or whole genome duplication (WGD) with subsequent gene loss can obscure synteny among related species [3]. Even though the placement of WGD is still controversial in the rosid lineage, it appears that *Vitis *(grape), which is a basal rosid, has undergone less WGD than *Populus *(rosid I) and *Arabidopsis *(rosid II), and *Arabidopsis *has undergone numerous additional chromosomal rearrangements [4-7]. We have previously reported a complex network of microsyntenic regions between *Prunus *and *Arabidopsis *using map-anchored *Prunus *sequences and *Prunus *BAC sequences [8,9]. The level of microsynteny between *Prunus *and *Populus*, however, was considerably high, reflecting the closer evolutionary distance between *Populus *and *Prunus *(Figure 1) and the apparent stability of the *Populus *genome compared to *Arabidopsis *[4].

The NBS-LRR family is the largest class of Resistance genes (R gene). In addition to the genetically cloned R gene loci, a large number of R gene analogs (RGAs) have been isolated from various plant species [10]. In grass, interspecific analyses have shown that the R genes are frequently found in non-syntenic positions unlike other genes, suggesting rapid reorganization of R genes [11]. In Solanaceae, however, conserved syntenic R genes have been described [12].

In this report, we show the microsynteny between *Fragaria *fosmid sequences [13,14] and other model genomes and also a cluster of NBS-LRR type genes that are conserved in all the genomes compared.

## Results and Discussion

### Microsynteny between *F. vesca *and other plant model genomes

To study the degree of synteny conservation between the strawberry and other sequenced plant model organisms, we used the 50 strawberry fosmid sequences [13,14] downloaded from NCBI. More than half (26 out of 50) of the strawberry fosmids contained microsyntenic regions in *Populus*, *Vitis*, *Medicago *and/or *Arabidopsis *(Table 1). Four syntenic regions were conserved in all five genomes and four were conserved in four genomes (Table 1). Twelve strawberry fosmids detected multiple syntenic regions in more than one chromosome of the same species, supporting the history of genome duplication events in these model species (Table 1). The microsyntenic regions between the strawberry fosmids and the model genomes contained three to seven gene pairs and covered 7 to 142 kb in model genomes and 7 to 35 kb in strawberry fosmids (Table 2). The number of conserved syntenic regions in *Vitis *was less than in *Populus *but more than in *Medicago *or *Arabidopsis *(Table 2). The *Medicago *genome is partially sequenced, so the number of microsyntenic regions may increase when the whole genome sequence data is used in the analysis. However, the degree of synteny between *Vitis *and strawberry was unexpectedly high considering Vitaceae is a basal rosid, the earliest diverging lineage of the rosids. The rate of gene loss after large genome duplication can vary depending on the species, and different rates of genome evolution may have been applied in these species after the ancient genome duplication. It could also be partly explained by the studies that suggest that *Vitis *has undergone less WGD than *Populus *and *Arabidopsis *[5-7].

**Table 1:** Microsyntenic regions between Fragaria fosmids and P. trichocarpa, V. vinifera, M. truncatula or A. thaliana

*F. vesca*	*P. trichocarpa*	*V. vinifera*	*M. truncatula*	*A. thaliana*
*01I13*	*VI (6); XVIII (6)*		*Chr3 (5)*	*Chr5 (4)*
01L02	scaffold_66 (3)			
05N03	VIII (3); X (3)	Chr1 (3)		
08G19	II (3)	Chr18 (3)		
12K04			Chr5 (3)	
10B08	X (3)		Chr3 (3)	
13I03	X (3)	Chr1 (3); Chr13 (4); Chr18 (3)		
*17O22*	*VII (3)*	*ChrUR (3)*		*Chr1 (3)*
19H07	XII (3); XIV (3);	Chr15 (3); Chr16_R (3)	Chr7 (3)	
19M24	XV (3)		Chr1 (3)	
30I24	VI (4); XVIII (3)			Chr4 (3)
**34E24**	**VII (4)**	**ChrUR (4); ChrUR (3)**	**Chr5 (3); Chr8 (4)**	**Chr5 (3)**
38H02	VII (3); scaffold_158 (3)			
40M11	II (3)	Chr18 (3)		
41O22	IV (5); XI (6); scaffold_64 (3)	ChrUR (3)		
48I08	X (4)	Chr13 (5)		
**51F10**	**scaffold_131 (4)**	**Chr14 (3)**	**Chr1 (4); Chr7 (4)**	**Chr5 (3)**
52B01	scaffold_123 (3)		Chr2 (3)	
52E09	XVII (3)			
*52I20*	*XII (4); XIV (3); XV (3)*	*Chr16_R (4)*		*Chr5 (3)*
*53J04*	*IV (4); XI (4)*	*ChrUR (3)*	*Chr2 (3)*	
53O08	V (3)	Chr18 (3)		
**72E18**	**VIII (7); X (5)**	**Chr13 (4); Chr6 (3)**	**Chr7 (4)**	**Chr3 (3); Chr3 (3)**
73I22	IX (3)	Chr13 (3)		
**75H22**	**XII (4)**	**ChrUR (5)**	**Chr1 (3)**	**Chr5 (4)**
76K13	II (4); V (3)	Chr18 (4)		

Numbers in parentheses are the number of gene pairs in the syntenic region.

Bolded are the syntenic regions that are conserved in all five genomes.

Italicized are the syntenic regions that are conserved in four of the five genomes

**Table 2:** Number and the length of the syntenic regions between Fragaria fosmids and the model genomes

	# of syntenic regions (# of fosmid with synteny)	# gene pairs (#syntenic regions)	Length* (*Model genomes*)	Length* (Strawberry)	Median E value
*Populus *	37 (25)	3 (22), 4(9), 5(2), 6(3), 7(1)	9.9 -- 86.3 kb	6.7 - 35.3 kb	1.00E-95
*Vitis*	23 (17)	3(16), 4(4), 5(3)	13.8 - 142.8 kb	6.6 - 32.4 kb	8.00E-98
*Medicago*	13 (11)	3 (8), 4(4), 5(1)	8.4 -- 55.5 kb	9.8 - 29.7 kb	2.00E-56
*Arabidopsis*	9 (8)	3 (7), 4(2)	7.1 - 11.7 kb	11.8 - 27.2 kb	1.00E-74

**Figure 2:**
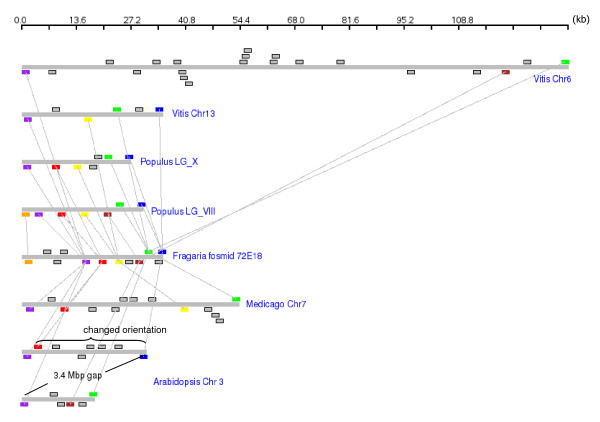
**Conserved microsyntenic regions with Fosmid 72E18**. The genomic segments are drawn according to their size but the genes are drawn with the same generic size. Squares above the lines represent genes located on the Crick strand and those below the lines represent genes on the Watson strand. Genes with the same color represent homologs and the gray squares represent genes without homologs in the genomic regions shown. *Vitis *Chr6, *Populus *LG_VIII, *Medicago *Chr7, and *Arabidopsis *genomic segments are drawn 3' to 5' and the rest are 5' to 3'. The diagram is drawn using GenomePixelizer [31].

A region in LG VIII of the poplar genome and the strawberry fosmid 72E18 had a syntenic block with the most gene pairs - 7 out of 12 genes in the fosmid were collinear. Since the syntenic region includes the first and last predicted genes of 72E18, the syntenic block can be potentially larger. 72E18 had another syntenic region in LG X of poplar and in all other genomes compared (Figure 2).

#### Detection of putative orthologs of NBS-LRR cluster

**Figure 3:**
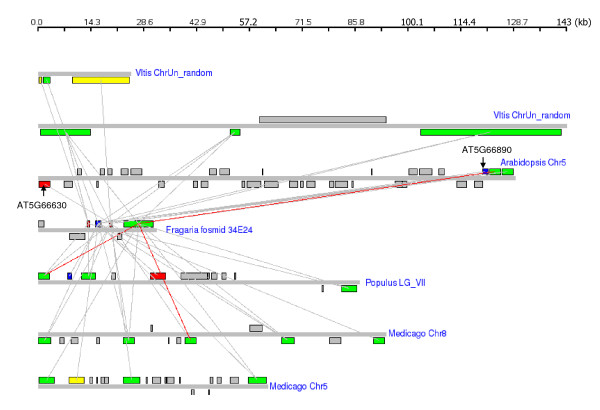
**Homologous RGA clusters in the five plant genomes analyzed**. Genomic segments and the genes are drawn according to their relative sizes. Squares above the lines represent genes located on the Crick strand and those below the lines represent genes on the Watson strand. The red line indicates the reciprocal best hit. Colored squares represent the genes containing R gene specific domains and the gray squares represent the intervening genes. The green squares represent genes with both NBS and LRR domains, the blue ones represents those with LRR, the yellow ones represents those with NBS, and the red ones represents those with fragmented NBS. All the genomic segments are drawn from 5' to 3'. The diagram is drawn using GenomePixelizer [31].

One of the fosmids, 34E24, had four RGAs and our synteny analysis found similar clusters in all the genomes compared (Figure 3). Multiple RGAs in the strawberry fosmid matched to multiple RGAs in the clusters of other genomes. Three genes in 34E24 are not related to R genes, and none of these genes had matches in the R gene cluster-containing regions of other compared genomes. 34E24_7, the longest predicted RGA in the strawberry fosmid 34E24, had reciprocal top matches in the syntenic region of *Populus*, *Medicago*, and *Arabidopsis *(Figure 3). 34E24_7 and its reciprocal top matches in *Populus*, *Medicago*, and *Arabidopsis*. 34E24_7 was the best match of the *Vitis *gene GSVIVP00003147001, and the *Vitis *gene was the second best match of 34E24_7. The observation that the homologous regions contained the reciprocal best hits and that they showed high percent identities suggest that these are putative orthologous regions. Since only RGAs in the genomic regions matched, however, it is possible these RGA clusters reside in non-syntenic positions.

InterProScan analysis showed that most of the genes in the clusters have the characteristic domains of NBS-LRR proteins [15] (Table 3). The NBS domain of NBS-LRR proteins is also called NB-ARC since it is composed of two contiguous sub-domains, NB and ARC [16]. The NB sub-domain contains kinase 1a (P-loop), RNBS-A, and other kinase motifs that are common to a variety of other nucleotide binding proteins. ARC sub-domain contains GLPL motif [16]. Studies have shown that there are two distinct groups of NBS-LRR genes, with or without the N-terminal Toll/Interleukin-1 receptor (TIR) homology region [12,17]. Since most of the NBS-LRR genes without the TIR domain have a coiled-coil (CC) motif in the N-terminal region [12], the two groups have been called TNL (TIR-NBS_LRR) and CNL (CC-NBS-TIR) [13]. None of the genes in the *Fragaria*/other genomes conserved cluster had a TIR domain, suggesting that they belong to the CNL group.

**Table 3:** Protein domains in RGAs and R genes that are predicted by InterProScan

Predicted Protein Domain	Species	Sequence Name
NB-ARC, LRR, RPW8, DISEASERSIST	*Arabidopsis*	AT5G66900
	*Arabidopsis*	AT5G66910
	*Fragaria*	34E24_7
	*Populus*	proteinId_563015
	*Medicago*	CU137666_10.3
NB-ARC, LRR, RPW8	*Vitis*	GSVIVP00003147001
NB-ARC, LRR, DISEASERSIST	*Populus*	proteinId_76151
	*Populus*	proteinId_76161
	*Medicago*	CU013515_11.5
	*Medicago*	CU013515_34.5
	*Medicago*	AC135229_1.5
	*Medicago*	AC135229_5
	*Medicago*	AC135229_9.5
NB-ARC, LRR	*Medicago*	AC148525_12
	*Medicago*	AC148525_17
	*Vitis*	GSVIVP00003149001
	*Vitis*	GSVIVP00003151001
	*Vitis*	GSVIVP00004841001
	*Prunus*	PP_LEa_1_Contig1043
NB-ARC, RPW8	*Vitis*	GSVIVP00004840001
	*Vitis*	GSVIVP00004842001
NB-ARC	*Medicago*	CU013515_31.5
LRR, RPW8, DISEASERSIST	*Populus*	proteinId_76154*
DISEASERSIST	*Fragaria*	34E24_5*
RPW8	*Fragaria*	34E24_3*
	*Arabidopsis*	AT5G66630*
LRR	*Arabidopsis*	AT5G66890
	*Fragaria*	34E24_4
	*Populus*	proteinId_563014

*Contains characteristic motifs of NBS domain

34E24_7 has two NB-ARC and three LRR domains. 34E24_7 also has two RPW8 domains in addition to these typical domains of NBS-LRR genes, one at the N-terminal of each NB-ARC domain. The *Arabidopsis *RPW8 gene, a representative of the most recently characterized class of R genes, is a small, probable membrane protein with no other homology to known proteins and it confers broad-spectrum mildew resistance [18,19]. We detected genes that have a similar structure to 34E24_7, containing NB-ARC and LRR domains in addition to RPW8 domains, in all the genomes compared: two *Arabidopsis *genes, AT5G66900 and AT5G66910, one *Medicago *gene, CU137666_10, one grape gene, GSVIVP00003147001, and one *Populus *gene, proteinId_563015. This M*edicago *gene has recently been reported as one of the NB-ARC genes with atypical domain structure due to the fused RPW8 domain [20]. We also detected two grape genes with both NB-ARC and RPW8 domains but without LRR domains. The majority of the R genes in the different species clusters had NB-ARC and LRR domains without RPW8, which is characteristic of the largest class of R genes.

**Figure 4:**
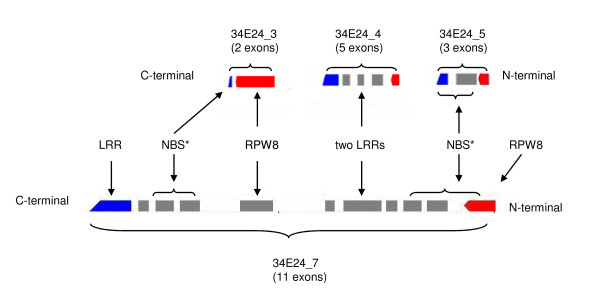
**Diagram showing the domain structures of 34E24_7 and the three nearby predicted genes**. The diagram compares the domain structure of 34E24_7 and three nearby genes, 34E24_3, 34E24_4, and 34E24_5. The domain structures were obtained by InterProScan analysis. 34E24_3 and 34E24_5 did not have the intact NBS domain but had some motifs characteristic of NBS domain.

The rest of the three RGAs in the fosmid 34E24 do not contain NB-ARC domains even though they matched to NB-ARC genes in other genomes (Table 3). 34E24_3 contains the RPW8 domain, 34E24_5 contains a disease resistance protein signature detected by the PRINTS database, and 34E24_4 contains a LRR domain. Close examination of these genes, however, revealed that they did contain some of the motifs that are characteristic of the NB-ARC domain. Both 34E24_3 and 34E24_5 contained a P-loop, and 34E24_5 contained RNBS-A and a kinase as well. The examination also revealed that the order of the domains in these three clustered genes, 34E24_3, 34E24_4 and 34E24_5, is very similar to that of 34E24_7, suggesting a potential gene duplication event followed by gene rearrangements to produce three smaller genes (Figure 4). The observation that four strawberry RGAs match to the similar sets of genes in other genomes (Figure 3), in spite of their different domain components, also suggest that these genes share common evolutionary history.

The *Arabidopsis *and *Populus *RGA clusters also contained genes with LRR or fragmented NBS domain without the intact NB-ARC: proteinId_76154 and AT5G66630 with fragmented NBS, proteinId_76154 with LRR and RPW8 domains, and AT5G66630 with a RPW8 domain. A previous study [15] has shown that AT5G66630 contains a zinc-finger domain and clusters with other zinc-finger domain containing genes, but it is fused with the NBS-like domain. The study also reports that the NB-ARC like domain of AT5G66630 is related most closely to a nearby cluster of NBS genes, one of which (AT5G66890) is lacking the NBS region, suggesting a translocation of this domain [15]. In our analysis, the RGA cluster in *Fragaria *matched to both the AT5G66630 and the nearby cluster of R genes including AT5G66890 (Figure 3).

One interesting observation was the occurrence of the LRR-only genes in the NBS-LRR gene clusters of several plant genomes. LRR domains are found in numerous proteins with various functions and are usually involved in protein-protein interaction [21] and they are considered to be responsible for R specificity [18]. Two classes of R genes, the tomato Cf-X genes [22] and the rice Xa21 [23], encode transmembrane proteins with extracellular LRRs. The frequent existence of the NBS fragments without LRR domains prompted a suggestion that they may encode adaptor molecules that are important in signaling [18]. Similarly, the existence of the LRR-only genes may suggest their functional importance in the disease-resistance mechanism involving NBS-LRR R genes.

#### Phylogeny analysis of the NBS-LRR genes in the clusters

**Figure 5:**
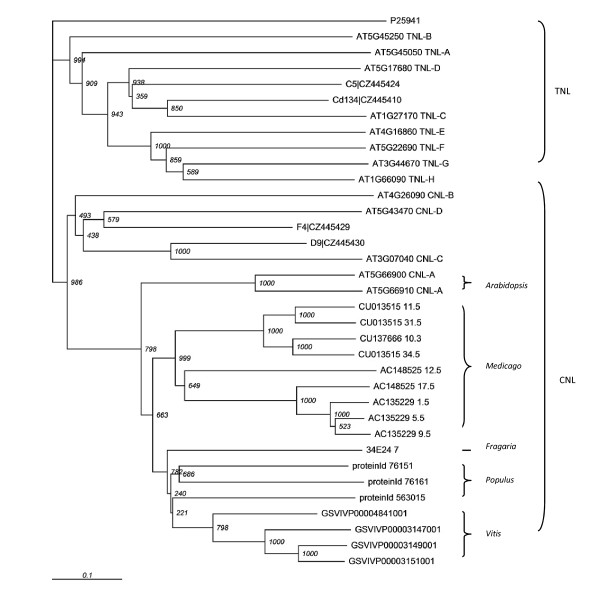
**Phylogenetic tree of the NBS-LRR genes**. The tree is rooted using Streptomyces sequence P25941 as an outgroup. The trees were drawn using TreeView [32]. The tree is divided into two major groups, TNL and CNL. All the homologs detected from this study group together with CNL-A, a subgroup of CNL. The *Arabidopsis *genes that belong to TNL (A through H) and CNL (B through D) are used as controls. The *Prunus *genes C5, Cd134, F4, and D9 are also used as controls.

To determine which NBS-LRR subgroup the genes we detected belong to, we performed phylogeny analysis on the genes that contain both NBS and LRR domains (Figure 5). In this analysis, we included the representatives of sub-groups of NBS-LRR genes [13] and some of the previously reported RGAs from *Prunus *genomic DNA [10]. All the CNL genes were grouped together separately from the TNL genes, and all the genes that we detected in our analysis grouped with other known CNL genes (Figure 5). The CNL branch was further divided by the subgroups, CNL-A, CNL-B, CNL-C and CNL-D, identified by a previous study [15]. All the NBS-LRR genes in the clusters that have been identified in our study belong to the CNL-A branch (Figure 5). In the CNL-A branch, genes from the same or related species, *Medicago*, *Arabidopsis*, *Vitis*, and *Populus*, grouped together (Figure 5). *Arabidopsis *genes in the CNL-A branch formed a separate basal group, and the rest was further divided into two groups, one with the *Medicago *genes and another with the rest (Figure 5). The group contained RGAs from *Fragaria*, *Populus *and *Vitis *(Figure 5). The observation that RGAs from *Vitis *group together with RGAs from *Fragaria *and *Populus *is in accordance with the unexpectedly high synteny between *Fragaria *and *Vitis*. The CNL-B, CNL-C, and CNL-D genes formed separated branches and did not contain any of the genes identified by our analysis. The non-TIR-type *Prunus *RGAs D9 and F4 belong to CNL-C and CNL-D, and the TIR-type *Prunus *RGAs Cd134 and C5 belong to TNL-C and TNL-D, respectively.

## Conclusions

We report the result from our comparative genomic sequence analysis of *Fragaria *and other rosids. Considerable microsynteny was detected between *Fragaria *and other plant genomes such as *Populus*, *Medicago*, and *Vitis*, and *Arabidopsis *to a lesser degree. The unexpectedly high level of synteny between *Fragaria *and *Vitis *and the low level of synteny between *Fragaria *and *Arabidopsis *suggest that the stability of genomes, in addition to the evolutionary distance, is important in synteny conservation. We also detected a cluster of NBS-LRR type R genes in all rosids analyzed in this study. The clusters included R genes with unusual domain structure such as NBS only, LRR only and NBS-LRR genes with RPW8. The phylogeny analyses showed that the NBS-LRR genes belong to CNL-A type.

## Methods

### Data Acquisition and Detection of Conserved Syntenic Regions

The 50 *Fragaria vesca *fosmid sequences [13,14], were downloaded from NCBI. Results of detailed analysis of the fosmids, including fosmid construction, sequencing, and identification of genetic elements are summarized in two publications [13,14]. We performed gene predictions using fgenesh and the *Medicago *(rosid I) trained gene set [Additional file 1], since the predicted gene sets [13,14] were not available at the time of analysis. The protein data of *Arabidopsis*, *Populus, Vitis*, and *Medicago *were downloaded from the web sites of TAIR [24], JGI [25], Genoscope [26], and http://www.medicago.org, respectively.

The predicted protein sequences of the *Fragaria *fosmids were compared with the *Medicago*, *Populus*, *Arabidopsis*, and *Vitis *proteins by pairwise comparison using the BLASTP program. The top ten matches with an E value less than 1e ^-10 ^were used for further analysis. Syntenic groups with at least three gene pairs were selected when the distance between the two adjacent matches were less than 200 kb, using DAGchainer [27], as described before [9].

### Detection of Domains and Phylogeny Analysis of NBS genes

The clusters of genes that matched to the cluster of genes in the *Fragaria *fosmid 34E24 were analyzed for known domains using InterProScan at the InterPro Database. The NBS-LRR proteins sequences were aligned using CLUSTAL W [28] with default parameters for slow/accurate option, available at Kyoto University Bioinformatics Center [29] and phylogenetic trees were generated using Neighbor Joining method. The NJ tree was bootstrapped (1000). The *Arabidopsis *sequences used as controls for various subtypes of TNL and CNL [15] and the *Prunus *RGAs [10] were downloaded from NCBI.

## List of abbreviations

RGA: Resistance Gene Analog; NBS: Nucleotide Binding Site; LRR: Leucine Rich Repeat; WGD: Whole Genome Duplication; BAC: Bacterial Artificial Chromosome; TIR: Toll-Interleukin Receptor; CC: Coiled Coil; RPW: Resistance to Powdery Mildew; TNL: TIR-NBS-LRR; CNL: CC-NBS-LRR; ARC: Apaf-1 R proteins, and CED-4; TAIR: The *Arabidopsis *Information Resources

## Competing interests

The authors declare that they have no competing interests.

## Authors' contributions

SJ conceived of the study, designed the protocol for synteny analysis, performed the research, analyzed the data and wrote the paper. IC wrote the scripts for parsing the DAGchainer outputs and set up the system to run various programs for data analysis and generating figures. BS and AA critically revised the manuscript. DM conceived of the study and participated in its design and coordination, and critically revised the manuscript. All authors read and approved the final manuscript.

## Supplementary Material

Additional file 1 **The predicted genes in 50 Fragaria fosmids**. The gene prediction is done using fgenesh program. The NCBI accession numbers of the fosmids and the start and stop position of the predicted genes are listed in the table. Click here for file
